# On the Causes and Morbid Anatomy of Mental Diseases

**Published:** 1855-04-01

**Authors:** John Webster

**Affiliations:** Fellow of the Royal College of Physicians.


### ON THE CAUSES AND MORBID ANATOMY OF MENTAL DISEASES.

BY JOHN WEBSTER, M.D., F.R.S.

*Fellow of the Royal College of Physicians.*

(Continued from p. 147, No. XXIX.)

No. 101.—M., æt. 52. In hospital eleven years and two months.—*Head:* Some serous fluid escaped on opening dura mater. Arachnoid covering cerebral hemispheres opaque, and accompanied in considerable degree with thickening in neighbourhood of great longitudinal fissure: here arachnoid surfaces slightly adhered. Near extremity of left posterior lobe, a brownish red adventitious membrane, rather larger than a crown-piece, closely adhered to dura mater. Cellular texture of pia mater infiltrated, and increased quantity of fluid in ventricles.

No. 102.—M., æt. 46. In hospital six months.—*Head:* Cerebral hemispheres flattened, especially anterior lobe. Arachnoid thickened, opaque, and of yellowish-white colour. In layers of pia mater covering hemispheres, large quantity of serum slightly tinged with blood effused. Convolutions atrophied. Cerebral substance moderately firm. Increased amount of serum in ventricles. Some small transparent cysts, containing limpid fluid, in spheroid plexus. Fornix softer than brain.—*Chest:* Both lungs extensively diseased, tuberculous, and broke down readily under pressure.—*Abdomen:* Entire large intestine congested, thickened, and presenting patches of ulcerated glands. Some parts of bowel converted into dark red structure, covered with thin layers of slough.

No. 103.—M., æt. 34. In hospital five days.—*Head:* Bloodvessels of cranium, membrane, and brain, all extremely turgid. Bloody points on cut surfaces of cerebrum large and very numerous. Arachnoid of moderate milky whiteness over whole hemispheres, and slightly thickened on convexities. Pia mater somewhat infiltrated, and adhered very firmly to convolutions. Left hemisphere of light murkish hue on surface. Lateral ventricles contained at least two ounces of limpid fluid; and much also remained at base of skull after brain was removed.—*Chest:* Pleurisy and pneumonia in left lung, with extensive mortification. Right lung partly hepatized, rest mortified, dark coloured, and emitted a most offensive odour, particularly from concave surface.

No. 104.—M., æt. 55. In hospital two months and three weeks.—*Head:* Skull-cap hard and brittle, though of normal thickness. Arachnoid sac contained more fluid than usual. Pia mater infiltrated with thick, and in some spots partly coagulated, serum. Ventricles distended by turbid serum. Fornix and septum lucidum reduced to pulpy state.—*Chest:* Heart small, and several white spots on surface of right ventricle. Black patches on pulmonary substance of left lung, which was soft, and emitted an offensive odour.

No. 105.—M., æt. 37. In hospital seven weeks.—*Head:* Dura mater very firmly adherent to bone. Vessels of both extremely turgid, and skull-cap heavy. Slight general thickening and opacity of arachnoid.—*Chest:* Left lung in congestive stage of pneumonia, dark, livid, and almost black coloured. Old adhesions of right lung, but less diseased than left.

No. 106.—F., æt. 45. In hospital thirteen years and five months.—*Head:* Cranial bones adhered firmly to dura mater, portions of latter being torn on removing skull-cap. Arachnoid partially opaque. Slight infiltration of pia mater. Cerebral substance soft. Lateral ventricles dry, and contained no fluid.—*Chest:* Trachea red, and bronchii congested. Lungs partially solidified, inflamed, and an abundant yellow coloured fluid, resembling pus, flowed from cut surfaces. Several puckered citatrices upon upper surface of right lung.

No. 107.—F., æt. 70. In hospital two months.—*Head:* Cranial bones paler than usual, and contained little blood. Dura mater purely white. Sac of arachnoid dry, and membrane perfectly transparent. Cerebral substance soft, and no fluid in lateral ventricles.—*Chest:* Old adhesions of pleura; right lung congested, and broke down under pressure. Bronchii inflamed.—*Abdomen:* Light fibrous tumours, from size of pea to a marble, attached to peritoneal surface of uterus.

No. 108.—M., æt. 45. In hospital three years and six months. *Head:* Dura mater adhered so firmly to bone, that it was torn in shreds by separating skull-cap. Arachnoid opaque, especially on longitudinal fissure. Pia mater moderately infiltrated. Cerebral vessels fuller than natural, and gave cut surfaces of brain a purplish hue. About an ounce and a half of fluid in lateral ventricles. Brain firm.—*Chest:* Traces of old tubercles in both lungs.—*Abdomen:* Ilium, colon, and rectum dark-coloured. Mucous membrane converted into soft, blackish-brown substance, and whole structure appeared partially disorganized.

No. 109.—F., æt. 67. In hospital twenty-six years and five months.—*Head:* Skull-cap of dull and somewhat livid colour throughout, from fulness of vessels. Bloody points on cut surfaces of brain conspicuous, and slight increased quantity of fluid in ventricles.—*Chest:* Space occupied by heart and pericardium twice usual extent. Serous effusion into pleural cavities. Lungs compressed and hepatized. Three or four ounces of serum in pericardium.—*Abdomen:* Liver small, and nutmeg-coloured. Gall-bladder large, and full of bile.

No. 110.—M., æt. 33. In hospital three years and five months.—*Head:* General fulness of external and internal vessels. Much blood escaped on dividing scalp and opening skull. Bone of dull red colour, dense, and heavy. Light serous infiltration of pia mater. Increased quantity of fluid in ventricles. —*Chest:* Heart and pericardium greatly increased in size, being equal to that of a bullock; and cavities much distended with coagula of blood. Mitral valves thickened, diseased, and edge ossified. Lungs condensed.

No. 111.—F., æt. 46. In hospital twenty-six days.—*Head:* Skull-cap thin and pale. Vessels of brain turgid throughout. Lateral ventricles large, but did not contain much fluid. Fornix raised, so that two cavities communicated by circular opening.—*Chest:* Heart large, and pericardium distended by considerable quantity of opaque, slightly yellow fluid, containing soft fibrine. Serous surfaces of pericardium covered universally with layers of fibrine.—*Abdomen:* Each ligament of uterus presented a cyst; that on right, the size of orange, the other of hen's egg; both filled with thick dark substance the consistence of tar, but not viscid, and considered blood. Two large and two smaller fibrous tumours in uterus.

No. 112.—F., æt. 58. In hospital eight months.—*Head:* Skull-cap heavy, yet easily separated from dura mater. Arachnoid transparent, but raised over whole upper surface of left hemisphere by extensive extravasation of blood in pia mater. Section of brain showed also considerable extravasation in substance. Thick black grumous layer of blood under tentorium cerebelli, extending along medulla oblongata and upper part of spiral chord, which were encased everywhere in clot. Right lateral ventricle contained much serous fluid. Upon surface of hemisphere, and into substance, smaller extravasation of black clotted blood were noticed.—*Chest:* Adhesions of both lungs to parietes. Heart hypertrophied, and left ventricle dark-coloured.—*Abdomen:* Old adhesions of peritoneum. Both kidneys atrophied, but left much more than right.

No. 113.—F., æt. 53. In hospital fourteen years and six weeks.—*Head:* Cerebral convolutions shrunken, leaving considerable spaces between, where pia mater was infiltrated with transparent straw-coloured gelatinous fluid. Whole substance of brain pale-coloured. Much serous fluid in base of skull after removal of brain. Choroid-plexus contained scarcely any blood.—*Chest:* Adhesions of lungs. Masses of tubercles, softened, and formed into cavities. —*Abdomen:* Large slough in groin, which entered through parietes to a pouch in iliac fossa that contained nearly three ounces of dark clotted blood. Cæcum adherent to parietes, whilst an opening, size of a pencil, communicated both with external slough and iliac fossa. Walls of ilium and cæcum thickened by tuberculous deposit. Canal so narrow, that a crow-quill could scarcely pass. Mucous membrane dark bluish-black coloured in various parts, and ulcerated. Cyst, size of a sparrow's egg, in right ovary, filled with coffee-coloured fluid.

No. 114.—F., æt. 80. In hospital forty-six years and eight months.—*Head:* Brain of moderately firm consistence. Convolutions atrophied, interspaces being filled by clear serous fluid.—*Chest:* Cavity of thorax much deformed. Adhesions of left lung to parietes.

No. 115.—M., æt. 43. In hospital five months and ten days.—*Head:* Brain bulged over sawn edge of bone. Cerebral vessels large, and full of blood; ventricles contained moderate amount of serous fluid. Thin layer of recently effused soft yellow lymph on middle fossa of skull; also cerebellar fossa, and upper process of basilar process. Dura mater, in these situations, greatly injected with blood.—*Chest:* Pericardium inflamed, which extended to left lung. Pulmonary substance universally dark coloured, and contained numerous circumscribed abscesses, some large as walnut, mortified, and emitted a strong odour.

### REMARKS.

Considered in the aggregate, the one hundred and fifteen dissections, described in the previous synopsis, clearly show that, in addition to decided morbid alterations of structure observed in the brain and its membranes of each case, ninety-nine patients, or more than four-fifths of the entire number, also manifested distinct marks of disease in one or more organs of the chest; whilst fifty-seven individuals, or half of the whole autopsies, likewise exhibited pathological changes, more or less extensive, in some of the abdominal viscera. Many of the cases I have now detailed are exceedingly interesting, and amply illustrate the morbid appearances usually met with in persons who died whilst labouring under mental alienation; although, in a large proportion of the cases described, the immediate cause of death seemed more owing to physical disease, especially affecting the thoracic contents, than to their attendant mental malady. Indubitably, the pathological appearances briefly detailed in the present communication would have proved much more instructive, had an outline of the chief symptoms, characterizing each patient's previous disease, been also appended, in order thereby to connect former psychical phenomena with subsequent morbid changes in the encephalon; and thus have endeavoured to point out, notwithstanding the known difficulty of all such attempts, how different phases of insanity more likely affect certain parts of the brain than others; in fact, to localize within that organ, if such were possible, the various forms of mania.

Considering this inquiry requires much greater experience than the present data yet supply, instead of pursuing such a difficult subject any further at present, I would now remark, respecting the pathological appearances met with in the one hundred and fifteen autopsies reported, that effusion of serous fluid into the ventricles was more frequently observed than any other morbid appearance; eighty-seven instances having been recognised. Infiltration of the pia mater ranked next in frequency; of which eighty-one examples are recorded. Turgidity of the bloodvessels of the brain and membranes follows afterwards, fifty-seven cases having been so enumerated. Besides the above instances of augmented serous effusion in the ventricles, thirty-four cases also showed an increased quantity of fluid collected at the basis craniid after the brain was removed. In twenty-eight patients, the dura mater adhered so firmly to the cranium, that this membrane was torn into shreds, and often very extensively, when separating the bone. Bloody points were noticed very numerously on the cut surfaces of the medullary substance in nineteen cases. The cerebral texture appeared firmer than natural in twenty-one persons; whilst in sixteen, the brain felt softer. In nineteen instances, the convolutions were shrunken; but on the other hand, in fifteen they seemed compressed or flattened. Effusion of blood, more or less copiously, had taken place into the cavities or texture of the medulla; whereby death, it may be hence reasonably assumed, was mainly produced. In eight individuals, the brain had become so enlarged in size for its containing cavity that, this organ bulged over the serous edges of the cranium, immediately upon the skull-cap being removed; whereas, in eight other cases, the bloodvessels were to a great degree, if not altogether, empty. Although not numerous, several examples, where the cerebral substance was altered in colour from its natural hue, were also recognised. Thus, in seven cases, the brain was changed to a pink hue, more or less pronounced; two appeared red; one was purple; one yellow; and in eight examples the medullary substance seemed much whiter than ordina y. Other important morbid alterations of structure, besides those now specified, are also noted in the synopsis, respecting which I would, however, only farther observe, before concluding this part of my subject, that in six autopsies the foramen of Monro was so much enlarged as to make a direct communication betwixt the two cavities.

Besides the above appearances, in a few cases an unusual dryness, and even a total absence of all moisture, between the cerebral membranes and its contents, was also remarked; and in several a soft yellow lymph had been effused, as if from recent inflammation; whilst in one interesting case (No. 101), a brownish-red adventitious membrane—the size of a crown-piece—adhered closely to the dura mater, covering left posterior lobe of the encephalon. Lastly, in another equally curious instance of morbid change (No. 18), the brain had become greatly decomposed in its substance, emitted an offensive smell, and had almost approached a state of mortification. The bony structure of the cranium likewise varied, in numerous patients, from its normal condition; upwards of one-third, the entire number being of that description. Thus, the skull-cap was found unusually thick and heavy in nineteen cases. In five, it appeared preternaturally thin. In four, the inner surface had become uneven, rough, and projected. In three, it was shallow. In two, the bone seemed so dense that the diploe had disappeared. In one instance, the calvarium was very hard and brittle, but of ordinary thickness; whilst in another case (No. 56), the clinoid process of the sphenoid bone appeared so prominent, as to project near half an inch beyond its fellow, being, however, smooth, and crusted with cartilage.

As already stated, a very large proportion of the one hundred and fifteen dissections contained in this communication, besides diseased changes in the cranial contents, also manifested decided morbid alterations of structure of the thoracic organs. Amongst the ninety-three cases so distinguished, eighty-one autopsies showed marks of recent or former inflammation of the lungs or their membranes. In thirty-nine cases, the heart or its valves were diseased. In twenty-three, the lungs were tuberculous. In twenty-three, collections of pus—some being large vomicæ—had formed. In nineteen, serous effusion had taken place into the pleural cavities; and in sixteen patients the lungs had become gangrenous, which alteration was in some cases even extensive; besides eight examples, wherein there appeared well-marked incipient signs of mortification. This large number of cases, in which gangrene of the lungs had actually supervened, constitutes a most important peculiarity in reference to maniacs; and seeing it coincides with the experience of other pathologists, the above facts, therefore, become more instructive. Irrespective of those individuals in whom evident indications of mortification seemed only to have just commenced, the ratio of one case of gangrene in about seven dissections, constitutes a very considerable proportion; and irrefragibly proves that, this form of diseased structure prevails much oftener amongst lunatics, than ordinary sick persons mentally sane. It likewise deserves special observation, that eleven of the cases occurred in males, whereas only five were females; which feature, therefore, conclusively indicates gangrene of the lungs as more likely to affect the former than latter sex, particularly patients whose mental malady was of recent occurrence; since all, excepting two, of the above sixteen cases, were parties who had not been insane during any length of time previously.

Believing it superfluous to discuss in detail, the various pathological appearances observed in the abdominal viscera of the fifty-four lunatics enumerated and coming within this division, I will only briefly remark on the present occasion that, in nearly one half, or twenty-five patients, inflammation of, or ulcers in, the intestines were noticed; whilst two cases of the latter category terminated in actual perforation; whereby fæcal matter had escaped into the peritoneum, and so caused death. In fifteen cases, the liver was diseased. In thirteen, inflammation of the peritoneum either recently prevailed, or adhesions of that membrane existed, but apparently of old date; whilst, in seven examples, serum was effused into the peritoneal cavity. Again, in eighteen female lunatics, the uterus was diseased, either from ulceration, the presence of fibrous tumours, or an affection of the ovaries; and lastly, in fifteen persons the kidneys appeared different, as to size or structure, from their natural condition.

Respecting several interesting peculiarities enumerated, in the general summary just given, of pathological alterations met with in the different bodies examined, I would here direct special attention to one or two features manifested by the above dissections, and from which some important practical inferences may be deduced. For instance, amongst the twenty-eight cases reported to have exhibited strong adhesions of the dura mater to the skull-cap—often so considerable as to cause that membrane to be torn into shreds, when removing the bone—twenty-two occurred in persons who had only recently become insane; the remaining six being chronic cases; whilst of these, seventeen were male, and eleven female, patients. Again, in the nineteen individuals whose skull-cap was thick and heavy, eighteen were recent examples of mental disease, with only one case which had been insane for a long period; amongst the former, seven being males, and eleven females. Of the five examples whose crania appeared preternaturally thin, four were recent cases, and one chronic; three being in males, and two in females. With reference to the altered consistence noticed in the brains of lunatics, whether firmer or softer than natural, it is important to mention that, of the twenty-one instances where the convolutions seemed unusually firm, thirteen were males, and eight females; whilst seventeen occurred in recent, and only four in chronic, cases of insanity. On the other hand, it should also be stated that, amongst the sixteen examples of softened convolutions, fourteen were recent, and only two were cases of long duration; one having actually continued an insane resident of Bethlem Hospital upwards of fifty-one years.

The additional point, to which I would lastly direct the notice of pathologists, is the proportion of cases where the convolutions of the brain seemed atrophied or shrunken. Seventeen such instances being recorded; of whom six were male, and eleven female, lunatics; eleven being in persons only recently insane, and six in parties who had been affected many years. Of the latter, three were females, one of whom had resided in the hospital upwards of fifteen years, one more than twenty-five years, and the third nearly forty-seven years: she being also in the eightieth year of her age. Again, as to the three males similarly affected, one had been an inmate upwards of six years, another more than twenty-nine years, and the last during thirty years. However, prior to deducing any inference respecting the greater frequency of shrunken or atrophied convolutions, in chronic than in recent cases of insanity, the fact should be always remembered that, as twenty-one dissections of the one hundred and fifteen now enumerated were made in patients who had been insane during many previous years, it hence follows, under one-third of those cases, which were of long duration, exhibited the morbid alteration now specified; whilst, among the remaining ninety-four autopsies of lunatics recently affected, only eleven examples, or nearly one-ninth of the aggregate number, showed similar phenomena. From such data it consequently appears that, old cases of insanity are more likely to exhibit shrunken or atrophied convolutions, than patients recently attacked.

Speaking generally, I may say confidently, many of the dissections contained in the synopsis now published are both instructive and interesting illustrations of the pathology of mental diseases. Two autopsies being however rather remarkable, as well in regard to the morbid changes of structure noticed after death, as also on account of their other features, therefore merit particular attention; more especially, seeing each case constitutes an example of suicide effected in an unusual manner; one, indeed, being almost without parallel in the annals of medicine. The first to which I would direct attention is No. 51 in the synopsis. This patient—a female—laboured under mania, and remained in Bethlem Hospital about one month. The present attack was not her first, having been insane about two years previously. She was suicidal, and had become recently again affected after the birth of a child. Was always hasty in temper, and although formerly of temperate habits, had recently, according to the report of friends, taken often to drinking; also fancied herself a person of title, and believed she ought to inherit considerable property. On admission, this lunatic was flighty, wild looking, restless, and likewise talked of destroying herself. Appeared slovenly, dirty, untidy in person; and subsequently became very violent, spiteful, and mischievous. These symptoms continued with little variation till nearly one week before death, when an attack of diarrhœa supervened. This complaint had become, however, so much alleviated by opium, calomel, counter-irritation, and saline medicines that, the evacuations soon appeared perfectly natural; but although there remained still much debility, the patient now seemed greatly improved in physical health. This condition continued even to within one day of her dissolution, notwithstanding the mind appeared greatly disturbed, whilst she likewise became more flighty, restless, and mischievous than previously. Nothing remarkable, however, occurred until the morning of decease; when, after rising from bed as usual, she walked across the gallery; but having there met one of the nurses, was immediately led back to her sleeping apartment. While being so conducted, she felt faint, and would have tumbled, had it not been for the support rendered by the attendant. The lunatic, however, rallied for a little, then began to ramble, talked incoherently, and in a few minutes afterwards fell dead without apparent suffering. In this insane patient, death was occasioned by pieces of a hair comb which she had, evidently with the intention of self-destruction, previously swallowed wholly unknown to any attendant, two bits of that female appendage having perforated the walls of the intestines, besides other portions of it also found in the ilium and cæcum, as already detailed in the synopsis.

The second case which I would now likewise bring under the reader's observation, is No. 15 in the list of dissections. This patient was a young man of good education, and who recently occupied the situation of clerk in a mercantile establishment. His friends reported the present attack was the first, that he did not seem suicidal, and had only recently become insane; whilst, the symptoms exhibited were, in every respect, those of decided melancholia. When admitted into Bethlem Hospital, the patient was very much depressed in mind, spoke of his ill-spent life and wickedness. Subsequently, he would often remain the entire day without taking any notice of other persons in the ward, or of passing occurrences; seldom talked, and seemed regardless of cleanliness or the calls of nature. In this apathetic condition he remained during five weeks; when, having first borrowed a common sewing needle from another inmate upon some frivolous pretext, he then thrust it into his left side, between the nipple and sternum, adjoining the fourth and fifth ribs; where, on a careful local examination, a small puncture was visible. Being soon afterwards visited by the resident medical officer, the sufferer stated what had occurred, and also acknowledged that his purpose in committing this act was to kill himself. On making inquiry respecting the symptoms, he further said, his heart quivered on being pierced by the needle, and that he still felt the instrument in that organ when breathing. For some time subsequently, the patient appeared free from pain; but very often became excited, refused to take food, and also appeared quite different from his former depressed condition, frequently talked at random, and continued restless during the night. Two days after the accident, general constitutional disturbance supervened; the pulse then became one hundred and thirty, his skin hot, and tongue white, the respiration being weak, and principally abdominal; he also moaned much, whilst the slightest pressure on any part of his thorax could scarcely be borne. Notwithstanding the small quantity of nourishment recently taken, no indication of physical weakness was apparent; the party could sit up, and even turned himself without any difficulty, or appearance of suffering, till the morning of his decease. During all that day, however, he continued very restless, being seldom ever quiet for a minute at a time; the pulse now became scarcely perceptible, but still reached one hundred and thirty, and was very feeble. About a quarter of an hour before death, he sat up in bed, swallowed some jelly, appeared in some degree even comfortable, and then calmly expired a few minutes afterwards. In spite of every effort zealously made by Mr. Lawrence to extract the needle in the first instance, as also by Dr. Monro and Dr. Wood to alleviate the consequent symptoms, all treatment proved wholly ineffectual, and this unfortunate patient died at the end of four days, after having pierced his heart and pericardium with the needle already mentioned, which had perforated the left ventricle, as described in the statement given of the morbid appearances.

Both the above cases are exceedingly instructive; not only in reference to their pathological aspects, but as further showing the necessity of constant superintendence, lest lunatics should intentionally injure themselves, even where no suicidal propensity had been suspected, or manifested previously. Too much surveillance can never be exerted towards insane patients: seeing, the greatest cunning and ingenuity is often employed to obtain possession of any instrument capable of inflicting bodily injury. The second case of suicide here reported, and in which the lunatic actually terminated his own mortal existence by a common sewing needle, is both curious and interesting, independently of its pathological importance. In fact, the chief circumstance described would almost seem to realize Shakespeare's poetic allusion to self-murder, in the well-known soliloquy of Hamlet, where the Prince says, "when he himself might his quietus make with a bare bodkin." This quotation from the great dramatist seems almost applicable to the tragic occurrence now detailed, and is remarkable; whilst I am led to fear, the case related is not a solitary instance, seeing the hackneyed quotation here given often takes hold of the excited imagination of persons in this country. That murder has even been perpetrated by adopting the mode described in the above case I can readily believe; indeed, an example perfectly parallel, both in regard to the instrument employed and the fatal result, occurred some years ago near Gottenburg, in Sweden, where, a M. de Lacroix murdered his first wife, and then a second he had afterwards married; both diabolical deeds being accomplished by the identical instrument now mentioned—viz., an ordinary sewing needle. Having been put on his trial, M. de Lacroix confessed, that in the dead of night, when the fair victims were asleep, he thrust a needle into the heart of each, whereby death almost immediately ensued; and as he carefully wiped away any blood which oozed from the slight puncture thus made, all trace of bodily violence was easily effaced. Such terrible tragedies as those last quoted, although not of pathological interest, being nevertheless, in many essential bearings, illustrative of a new mode whereby the life of a fellow-creature is sometimes suddenly cut short by criminals, or by insane persons upon themselves, I trust this digression, notwithstanding it may perhaps appear to some readers rather foreign to the present discussion, will not be considered wholly irrelevant.

Before bringing these remarks to a close, I would further repeat that, the following is an epitome of the morbid appearances met with in the one hundred and thirteen dissections now communicated. In eighty-seven patients who died insane, effusion had taken place into the ventricles. In eighty-one, the pia mater was infiltrated. In fifty-seven, turgidity of the brain and membranes was observed. In fifty-five, the arachnoid coat had become thickened and opaque. In nineteen, the colour of the brain appeared altered from its natural hue. In nineteen cases, also, bloody points were both large and numerous upon the cut medullary surfaces; whilst, in ten instances, blood was effused—even sometimes to a considerable amount—within the cranium, and evidently acting as the immediate cause of death in these patients. According to the above summary it therefore appears, first, that effusion of serum into the ventricles, secondly, infiltration of the pia mater, and thirdly, turgidity of the bloodvessels of the brain or membranes, are the chief and most common diseased alterations of structure, which pathologists may confidently anticipate in the great majority of patients who die whilst labouring under mental alienation.

### Appendix.

Since the previous communication was written, several additional autopsies have been made at Bethlem Hospital, which I would therefore append. Like those already detailed, they possess considerable interest; and as a short statement of the prominent symptoms observed during their former mental malady is also added, these dissections therefore become more worthy of perusal. In the synopsis similar details did not accompany each case, chiefly because my communication might have thus extended to a greater length than seemed desirable. Such omissions indubitably diminished the value of various narratives then given, but which does not characterize those I have now subjoined. As already remarked, however useful it would become for practical physicians to know, beforehand, the precise portion of an insane patient's brain which may be affected by morbid changes of structure, when particular mental phenomena prevail during different forms of mania, hitherto, not much progress has been made in this department of psychological knowledge. Nevertheless, being anxious to promote, even partially, so important an inquiry, I am now induced to supply the following short contribution, although it may, perhaps, seem to contain, in some respects, rather limited information.

No. 116.—M., æt. 48. In hospital thirty-nine days.—*Head:* All the vessels of brain and membranes filled with blood to minutest ramifications. Slight partial opacity of arachnoid, and some infiltration of pia mater. Fluid of ventricles rather beyond normal quantity. Effusion of blood in cerebral fossæ of basis of cranii, sufficient to cover thereby lobes of cerebellum, and to line corresponding part of arachnoid with a layer of coagulum. Similar effusion, but to much less extent, on a small part of each cerebral hemisphere.—*Chest:* Posterior portion of left lung highly congested, and contained but little air.—*Acute Mania:* Very violent on admission, being brought to Bethlem confined in a strait-waistcoat, and with fastenings on his legs. Was exceedingly incoherent; frequently alluded to the war with Russia, and that he would kill the enemy. Speaks of his own great personal strength, butts his head against objects, kicks, strikes, and attempts to bite other persons indiscriminately. Is most destructive of his clothes, and even destroys in a day three or four suits of canvas; always very dirty, and continued nearly constantly most incoherent. Latterly the patient became weak, took food very indifferently, having scarcely strength to swallow a single teaspoonful of any liquid, even of brandy, which was ultimately the only kind of support or nourishment taken, until he died quite exhausted.

No. 117.—M., æt. 55. In hospital five weeks.—*Head:* External vessels empty, internal full of blood. Very slight infiltration of pia mater. Bloody points on cut surfaces of cerebral substance.—*Chest:* Masses of tubercles in a crude state scattered through lungs ; both divisions being also affected with recent pleuro-peripneumony, and which on posterior aspect was rather extensive. —*Melancholia:* Insane two months prior to admission, being often in a state of decided melancholy; refused food, and exhibited signs of committing suicide. When admitted, was much depressed in spirits, seemed very miserable, and soon afterwards appeared as if almost imbecile. Often stands helpless-looking in the ward, is frequently groaning, rarely if ever speaks, and never answers any question. Takes food with great reluctance, is frequently very restless at night, and sometimes exhibits nearly entire unconsciousness, which continued, with the other symptoms, till his malady terminated fatally.

No. 118.—F., æt. 37. In hospital ten days. On viewing patient's body, left breast was enlarged and livid; discoloration being at first supposed to have proceeded from effused blood, but it was found, on more minute examination, to be a considerable abscess, full of discoloured pus.—*Head:* External vessels quite empty, internal moderately full of blood. Pia mater infiltrated with perfectly limpid fluid in intervals of convolutions. Increased quantity of fluid in ventricles, with much also in and about velum. Substance of brain soft in central parts.—*Chest:* Old adhesions of right lung. Lower and back part of lower lobe of left highly congested.—*Acute Mania after protracted lactation:* Insane one week prior to admission, being then frequently violent, incoherent, and often swearing, although previously correct both in language and conduct. When admitted was very weak, blanched in countenance, and appeared to have suffered from very violent maniacal excitement, without her strength having been supported by sufficient nourishment. After admission, felt great reluctance to take food, which aversion soon became so marked that, afterwards she had to be almost forced to swallow whatever passed her lips. Subsequently, the poor sufferer became too feeble to be noisy, but continued always very restless, and did not remain a moment in one position. Having constantly great objections to take, not only solid, but even any liquid food or drink, she was therefore forced to swallow beef-tea and wine as support. Ultimately, the patient got very feeble, her pulse being scarcely perceptible; and at last she expired in a state of complete exhaustion.

No. 119.—F., æt. 45. In hospital nine days.—*Head:* Skull-cap thin and pale. Dura mater quite detached from bone. Substance of brain softer than natural, and septum lucidum broken through.—*Chest:* Adhesions of left lung, which was dark-coloured, and full of blood in posterior parts. Heart flabby, and of yellowish colour.—*Acute Mania:* Insane five months prior to admission, a brother being also affected with mental disease, and her malady reported to have been augmented by attending several theatrical performances, and she threatened to injure relatives. On admission was incoherent, very excitable, often excessively violent, and rambled in conversation. These symptoms continued without much abatement during the time this patient remained in hospital, and till the morning of her decease, when she was found dead in bed, as if asleep, but lying in a position indicating perfect repose. There appeared no contortion of countenance, and the body was pallid.

No. 120.—F., æt. 22. In hospital six weeks. Corpse extremely emaciated, and the whole surface, both of trunk and limbs, exhibited a dusky red or blackish hue. *Head:* Membranes of brain very full of blood. Dura mater of a light pink colour. Vessels under arachnoid full of blood. Numerous dark spots on cut surfaces of cerebrum. Brain rather above normal consistence, and about a dram of serous fluid in ventricles.— *Chest:* Both lungs contracted towards posterior part of cavity. Muscular substance of heart discoloured, the whole organ being soft, flabby, and contained a quantity of dark fluid blood.—*Abdomen:* Viscera soft, and in partial state of decomposition. Entire ovarian apparatus appeared shrunken and bloodless. *Dementia from Masturbation:* Father and brother of this patient were also insane. She has attempted suicide, and has laboured under mental disease during seven months prior to admission. Is said to have eaten her excrements, often tears her clothes, and frequently gets violent from religious excitement. Having been brought up with the expectation of inheriting a fortune, she became much depressed when reduced to poverty. Soon after this unfortunate event, symptoms of mental aberration first appeared. On admission, her person was thin and emaciated, habits filthy and indecent, whilst no feelings of modesty or delicacy prevent her indulging anywhere, or at any time, in those bad propensities to which she is addicted. Occasionally talks rationally, but very soon afterwards thinks the devil is going to take her to hell; appetite is voracious. Sleeps well. Soon afterwards became much more excited and restless than before, as likewise more dirty. Required being carefully watched to keep her in bed, as she bruised herself from rolling about and by constant contortions. Ultimately, extreme debility supervened, and she became quite exhausted previous to death.

No. 121.—M., æt. 44. In hospital eight years and nine months.—*Head:* Vessels of brain and membranes distended with blood. Pia mater moderately infiltrated. Increased quantity of fluid in ventricles.—*Chest:* Right lung full of small tubercles, with also numerous vomicæ. Left lung attached extensively to parietes by strong adhesions; unattached portion being covered by a thick stratum of whitish tolerably firm fibrine, not organized. A few ounces of fluid in cavity. Posterior part of upper lobe in congestive state of inflammation. Lower lobe partially consolidated. Pericardium closely adherent to heart throughout entire surface; membrane being considerably thickened and indurated.—*Abdomen:* Colon descended to brim of pelvis, and was in contact with symphisis pubis, it then passed to left hypochrondrium, from whence the intestine afterwards pursued its regular course.—*Melancholia:* Disease hereditary; his grandfather having been also insane. Is suicidal, and has attempted to drown himself. When admitted, believed all food given him was human flesh and blood, that he formed one of the Trinity, and therefore, had become a divine personage. Mind weak, if not imbecile; and although rather clever as a designing artist, when asked to employ himself in any useful manner often replied: "If sufficiently well to work, I am, therefore, well enough to be set at liberty." This patient continued much in the same state till his death, which ultimately arose from pectoral disease as shown by the autopsy.

No. 122.—M., æt. 34. In hospital eleven days.—*Head:* Brain firm, and vessels somewhat fuller than natural. Several opaque spots on arachnoid. Cerebellum softer than natural, and lay in a quantity of turbid serous fluid, which seemed to fill whole vertebral canal.—*Chest:* Left lung slightly adherent at upper portion, and presented a dark bluish-black hue, over whole posterior and lower surface. Cut surfaces nearly black, and structure friable; organ in last stage of congestive pneumonia, and reduced, in many parts, to a dark brown coloured semi-fluid substance of offensive odour. Right lung extensively mortified. About a pint and a half of serous fluid in pleural sac.—*Abdomen:* Gall bladder distended. Small intestines, dark coloured, with a thick black fluid matter effused on mucous membrane, and resembled that which could be squeezed from disorganized lung.—*Mania:* Insane ten days prior to admission, but had been similarly affected on two previous occasions. Is now exceedingly restless, and labours under great excitement, although in a very debilitated physical condition. Seems quite regardless of the calls of nature. Particularly obstinate, and not only refuses all solid food, but even to take any liquid whatever, unless with the greatest difficulty. When the patient's name is pronounced sharply, he sometimes moves his head slightly, as if recognising the sound; but he can never be made to speak. Bowels very constipated. Two days after admission, during an attack of great excitement, had contortions of limbs, became very restless, could scarcely be kept in bed, shouted much, and used most indecent language, his pulse being also at this time almost imperceptible. Next day, he appeared quite unconscious, and would scarcely take nourishment. Although labouring under pneumonia, it was only towards the latter days of patient's life that any symptoms of pectoral disease seemed to supervene, a dulness being then discovered on percussion over the posterior part of right lung. He had no cough, and appeared altogether free from pain in chest.

No. 123.—M., æt. 27. In hospital six years and thirty weeks.—*Head:* Brain firm and white. Anterior lobes flattened. Supra-ventricular mass of cerebral matter below average in quantity, and shrunken.—*Chest:* Pericardium contained large amount of turbid serum; also a considerable quantity of serous fluid in pleural cavities. Left lung studded with opaque softened tubercles. Adhesions of right lung, and a large irregular cavity in substance.—*Abdomen:* Two tubercles in ilium. Mesenteric glands larger than natural, with serous fluid effused in peritoneal cavity. Kidneys small and flat.—*Melancholia:* Incurably insane for some time prior to admission; disease being originally caused by over study, and reported a suicidal patient; seems exceedingly depressed and dull, is weak in body, and much out of health. Has a rather prominent eye, which he generally keeps directed downwards. Appears very timid; avoids notice by other patients, and seeks retired corners, as if anxious to hide himself from observation. Does not relish any kind of employment, and is disinclined to every species of bodily exertion. Exceedingly restless at night; and takes food indifferently. Is reported to have made frequent attempts to strangle himself, as also to cause death by swallowing stones. Believes he has been unjustly excluded from all religious society, the people about him being quite changed, and that his mother is Catherine de Medicis. Before decease, became greatly emaciated.

Believing it superfluous to make lengthened commentaries upon the eight autopsies now appended, I will therefore only remark that, they constitute instructive illustrations of the varied forms of insanity usually met with in practice. Instances of violent mania have been thus recorded, as also of melancholia. Mania following protracted lactation, and dementia from masturbation being likewise described. Besides these examples, the interesting case of gangrene of the lungs will repay perusal; more particularly, since scarcely any symptom of the extensive pectoral disease actually existing could be distinctly ascertained during life, but which peculiar feature often characterizes this morbid change of structure as observed amongst insane persons, if compared with ordinary patients. Lastly, the case of chronic melancholia, with which the present series concludes, specially merits notice, seeing it furnishes another illustration to those previously given, of a rather common occurrence in persons long insane—viz., where the cerebral matter appeared below an average quantity and shrunken.

---

